# Development and Testing of a Fully Adaptable Membrane Bioreactor Fouling Model for a Sidestream Configuration System

**DOI:** 10.3390/membranes3020024

**Published:** 2013-04-24

**Authors:** Parneet Paul

**Affiliations:** School of Engineering and Design, Brunel University, Uxbridge, Middlesex UB8 3PH, UK; E-Mail: parneet.paul@brunel.ac.uk; Tel.: +44-1895-265-435; Fax: +44-1895-274-000

**Keywords:** wastewater treatment, membrane bioreactor, modelling, time series analysis

## Abstract

A dead-end filtration model that includes the three main fouling mechanisms mentioned in Hermia (*i.e.*, cake build-up, complete pore blocking, and pore constriction) and that was based on a constant trans-membrane pressure (TMP) operation was extensively modified so it could be used for a sidestream configuration membrane bioreactor (MBR) situation. Modifications and add-ons to this basic model included: alteration so that it could be used for varying flux and varying TMP operations; inclusion of a backwash mode; it described pore constriction (*i.e.*, irreversible fouling) in relation to the concentration of soluble microbial products (SMP) in the liquor; and, it could be used in a cross flow scenario by the addition of scouring terms in the model formulation. The additional terms in this modified model were checked against an already published model to see if they made sense, physically speaking. Next this modified model was calibrated and validated in Matlab^©^ using data collected by carrying out flux stepping tests on both a pilot sidestream MBR plant, and then a pilot membrane filtration unit. The model fit proved good, especially for the pilot filtration unit data. In conclusion, this model formulation is of the right level of complexity to be used for most practical MBR situations.

## 1. Introduction

The focus of this research was to create practical membrane bioreactor (MBR) computer models that can then be applied to MBR plant design, control and optimisation. It was intended that the outputs of this research would lead to both the improvement of existing models and the creation of new, innovative models. The eventual application of this model type would be to optimise a real treatment plant and thereby eventually develop a long term energy saving control strategy. 

### 1.1. Previous Modelling Studies of MBR Fouling—Sub-Critical to Supra-Critical Flux Ranges

Mathematical modelling of the membrane fouling process can assist the engineer in improving the membrane operation and performance, *i.e.*, better prediction of the fouling rate and optimisation of the corresponding retaliatory actions needed to reduce it. For a MBR system treating wastewater, capturing membrane fouling phenomena in the form of mathematical models has been a task of many different global research teams over the past two decades. Most researchers model the membrane fouling process using a phenomenological mechanistic approach. In Le-Clech *et al.* [1], it was suggested that a “Three-Stage-Fouling” model should be considered for the total membrane fouling and clogging process for the entire flux range from sub-critical up to supra-critical fluxes. Based on experimental observation, the three stages of fouling are initial passive conditioning; slow and steady fouling (usually at sub-critical fluxes); and a rapid trans-membrane pressure (TMP) jump (usually at supra-critical fluxes, although not exclusively).

Under normal plant operation, supra-critical fluxes should not usually occur for the plant as a whole, although they will occur at a localised level for individual membrane modules or portions of modules due to localised uneven membrane blocking and clogging events. So any useful fouling modelling should be capable of catering for this. Cho and Fane’s area loss model [2] and the subsequent Ye *et al.* pore loss model [3] both consider that the sub-critical flux is exceeded at a local or regional level for certain portions of the membrane. These localised supra-critical fluxes then further reduce the area/pores contributing to sub-critical fluxes, and when they reach a critical tipping point, the dramatic TMP increase occurs. Chang *et al.* [4] developed an alternative fouling model based upon the critical suction pressure, where the model describes the sudden collapse of the cake layer under a critical TMP. This cake material can be highly compressible depending on the hydrodynamic regime employed and the cake composition (*i.e.*, floc size and structure). This sudden cake compression causes a rapid build up in resistance in a very short space of time, and would explain the TMP jump experienced in practice. Hermanowicz [5] suggested and modelled an alternative clogging and fouling mechanism based on percolation theory in which the developed model uses the critical loss of diffusivity of the cake layer to explain the TMP jump stage. In this model, the cake layer is taken as being highly porous with smaller particles, aggregates and colloidal material travelling towards the concentration polarisation zone by natural convective transport, and taking up the void spaces in the cake layer which would be currently taken up by fluid. Hermanowicz [5] theorised that when this mechanism reaches a critical point, the cake resistance increases dramatically, causing the apparent TMP jump.

Over that time many approaches of various complexities and usefulness have been adopted to explain foulability [6], with some research groups developing models that describe the entire MBR plant configuration and operation [7]. For instance Broeckmann *et al.* [8] developed a fouling model for a MBR system that also considered pore size and floc size distributions which are an important aspect in contributing to overall fouling potential. Busch *et al.* [9] developed a fully comprehensive model which consisted of several sub-models that modelled different aspects of the MBR process. These models could then account, for example, for membrane configuration and geometry (e.g., hollow fibre, flat sheet, multi-tubular, *etc.*), the hydrodynamics of the feed flow, the hydrodynamics of the permeate flow, the cleaning mechanisms employed (e.g., backflushing, air sparging, relaxation, *etc.*), and the various filtration resistance components. Gehlert *et al.* [10] created a rigorous mathematical model of an open channel cassette module for use in a MBR. In contrast, Zarragoitia-Gonzalez *et al.* [11] created a mathematical model to simulate the filtration process, and aeration influence on a submerged MBR operated under aerobic conditions. In contrast Lee *et al.* [12] developed a very simple cake build up model which just considered the mixed liquor suspended solids (MLSS) in very simple terms. Similarly Wintgens *et al.* [13] produced an easy-to-use cake build up and pore blocking model based upon a simple resistance-in-series expression, which however didn’t reflect reality that well as it depicted total membrane resistance as reaching a saturation value at supra-critical fluxes when in fact in practice it is an exponential increase when the TMP jump occurs. This does not mean to say that a simple but relevant model can never be developed, since Ognier *et al.* [14] did just this by introducing the concept of local critical flux to explain the TMP jump conditions experienced at long filtration periods when no cleaning-in-place (CiP) procedures are applied. Ye *et al.* [15] developed a very similar but slightly more complex model for predicting TMP evolution under long term sub-critical filtration of synthetic extra-polymeric substance (EPS) solutions. In an alternative vein, Psoch and Schiewer [16] constructed a model based on pore constriction resistance, cake resistance and clean membrane resistance with air sparging and backflushing mechanisms also included.

Several classical fouling studies [17] use a three mechanism model for the bio-fouling process made up of pore constriction, pore blockage, and cake filtration. These mechanisms can be directly related to the main bio-fouling processes observed in a MBR system. This set of models uses the classical blocking laws developed by Hermia [17] as the start point for developing a model description. Ho and Zydney [18] developed the first real combined fouling model which accounted for the classical complete pore blockage equation, intermediate pore blockage equation and cake filtration mechanisms. The model was validated and was in good agreement with flux decline data obtained from bovin serum albumen (BSA) filtration experiments. This model was further extended by the extensive and comprehensive work of Duclos-Orsello *et al.* [19] in which an internal pore constriction mechanism was modelled as a reduction of pore diameters by the Hagen-Poiseuille Law.

### 1.2. Some Problems of Using Phenomenological Membrane Fouling Models for Plant Design, Operation and Control

Many of the earlier described traditional phenomenological membrane fouling approaches that are used to model MBR systems often suffer from some of the following interrelated problems and disadvantages:

1. As membrane fouling is in reality a very complex and very little understood process at this moment in time, it is difficult to make a generalised mechanistic fouling model that can adequately address all issues and specific nuances involved.
2. Ideally fouling models of this type need to be made bespoke for each individual filtration system on a case-by-case basis. This is especially true for the hydrodynamics of the process (e.g., type of sparging system or membrane scour system in use), and the membrane operational regime (e.g., submerged or sidestream or vertical air-lift).
3. The models are often highly dimensional and contain numerous parameters that need determination by specific plant data sets, specific process operations (*i.e.*, flux stepping trials) and with the use of extended specialist laboratory experiments (e.g., specific cake resistance tests). Thus the models, themselves, can be over-parameterised with too many degrees of freedom.
4. Parameter estimation and optimisation can prove to be a convoluted and complex procedure requiring expert knowledge and extensive experience.
5. For many applications insufficient data is available to allow a full model calibration and validation, and thus the verified model is not omnipotent for every situation.
6. The general application of such complex models, which in themselves require considerable calibration experience to give sufficient predictive accuracy, means their take up for process control and the development of future operational strategies will always prove limited [20].

The main research question posed in this study was how easy is it in practice to calibrate and validate a phenomenological membrane fouling model for a real life MBR plant which is rich enough in complexity to express the major fouling mechanisms involved. Thus in order to address this question and to overcome the inherent deficiencies in this traditional approach, a MBR fouling model was developed that was simple enough to express the key membrane filtration and fouling processes for a MBR whilst still having sufficient degrees of freedom so it could be used for model predictive control and plant operational and design purposes.

## 2. Description of Model Used—Duclos-Orsello

As stated previously several classical fouling studies use a three mechanism model for the bio-fouling process [17,18]. The Duclos-Orsello [19] model was chosen under this study as it contains all three main fouling mechanisms, and is sophisticated enough with sufficient degrees of freedom whilst still being relatively simple in structure with a limited number of model parameters requiring calibration. In the original model, Duclos-Orsello [19] splits the total flow, *Q_t_*, through the membrane into flow through the unblocked membrane surface area and flow through the blocked membrane surface area as shown in Equations (1) and (2). Hence the first algebraic term relates to the unblocked flow whilst the second integral term relates to blocked flow.



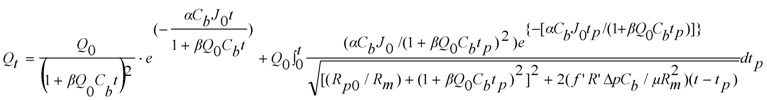
(1)


### 2.1. Practical Limitations of the Duclos-Orsello Approach

The following limitations were found in the Duclos-Orsello [19] approach:

1. Since Duclos-Orsello [19] uses solutions from solved differential equations when calculating the flow through the unblocked membrane area, the state variables in the study must remain constant during any simulation. In a real life plant this is not the case, with say the MLSS concentration which is analogous to the *C_b_* parameter (bulk concentration) in this model, often changing significantly in time. So any altered model formulation procedure must allow for this fact.
2. Since Duclos-Orsello [19] uses an integral expression to calculate the flow through the blocked membrane area, the initial conditions in this study must remain fixed in any simulation, and any simulation must always commence from this point. In any useful simulation model, the user must be able to state different and varied initial conditions, and also be able to start a simulation from any point in time. So any altered model formulation must cater for this modelling issue.
3. The integral expression means the TMP is kept constant during the simulation, and therefore the mathematical formulation of this model cannot be used for varying TMP situations which occur in many real life plant scenarios.
4. This model formulation assumes pore constriction stops as soon as pore blockage occurs. This may not be the case as experienced in real life membrane fouling within a MBR system.

### 2.2. Initial Improvements to Duclos-Orsello’s Approach

A summary of the re-modified equations with additional terms is provided below. When calculating the flow through the unblocked and blocked membrane areas, the model is formulated to use the original differential equations so that any state variables can be varied in time. This altered model formulation also has the further benefit of allowing parameter sets to be optimized using measured data values. Hence the change in flow rate through the open pores, *Q_u_*, in the reducing unblocked membrane area, *A_u_*, is calculated from Equation (3). This reduction in unblocked area is calculated from the differential Equation (4) where the unblocked flux, *J_u_*, is itself determined from differential Equation (5).



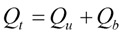
(2)




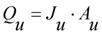
(3)




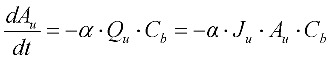
(4)




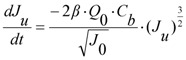
(5)


The change in flow rate through the closed pores, *Q_b_*, in the increasing blocked membrane area, *A_b_*, is calculated from Equation (6) which is based upon Darcy’s Law. This increase in blocked area is calculated from the differential Equation (7) where the rate of change in blocked area is equal to but negative in size of the rate of change in unblocked area.



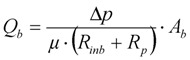
(6)




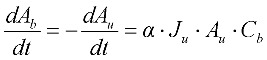
(7)


The resistance of the membrane in Equation (6) is made up of the initial blocked membrane resistance *R_inb_*, and the growing external cake layer resistance *R_p_*. *R_inb_* is calculated from Equation (8) where *R_m_* is the clean membrane resistance, whilst *R_p_* is determined from the differential Equation (9) where *f*′ is the fraction of foulants in the mixed liquor that cause the build up of the cake layer and *R′* is the specific protein layer resistance.



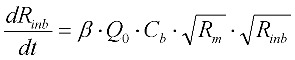
(8)




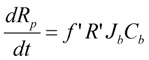
(9)


It has been observed in practice that membrane pore constriction continues even after solids cake build up in a MBR system [21] although the cake layer porosity determines the rate of this pore constriction after cake-layer build-up. This is because in real life the range of particle size distribution of the mixed liquor particles and colloids is very large as well as the pore size distribution of the membrane pores themselves. This means smaller colloids can be transported through the poorly packed porous cake layer into larger pores even though they may be partially occluded by larger particles. Hence the blocked membrane resistance, *R_bl_*, in this modified formulation is also composed of the pore constriction resistance which will continue even after the pore is blocked. *R_bl_* is calculated from Equation (10) where the continuing pore constriction is added to the initial blocked membrane resistance, *R_inb_*. The original model formulation of Duclos-Orsello [19] does not allow this process to happen.



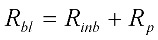
(10)


The final modified and improved version of the Duclos-Orsello [19] approach can be summarised by Equation (11) which determines the total flow through the membrane while all three mechanisms take place.



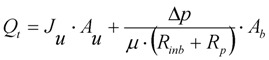
(11)


This new model formulation should allow it to be used for practical purposes such as for predicting the fouling rate on a real life MBR plant, and for the operation and automatic control of the plant. However any new MBR model would also need to include a plant layout with hydrodynamic regime, and would need calibration with actual measured data values.

### 2.3. Using SIMULINK^©^ Genetic Algorithm Toolbox to Optimise Model Parameters Using Duclos-Orsello’s Original Data

**Figure 1:**
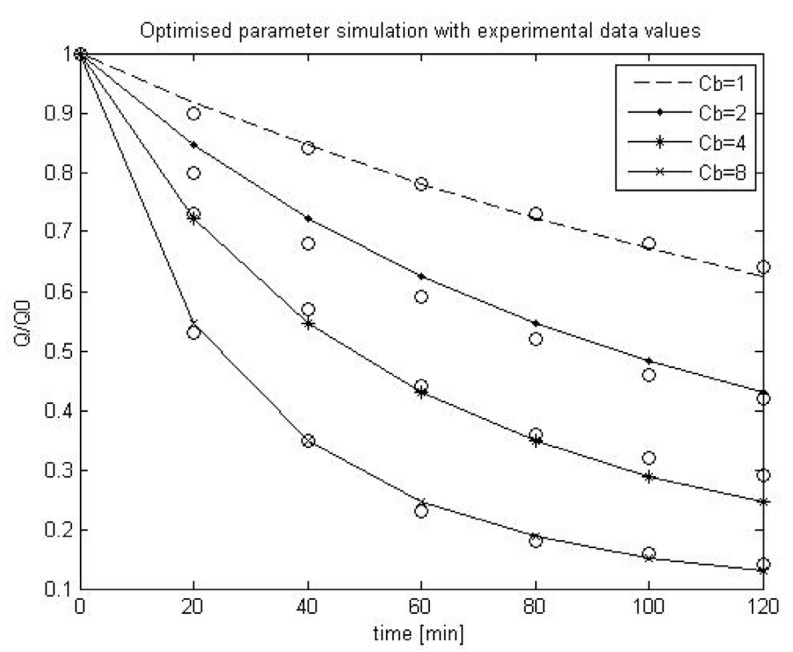
Comparison of optimal parameter simulation plot with experimental data values from the Duclos-Orsello paper [19].

The accuracy of this modified approach was confirmed using the experimental data from the Duclos-Orsello paper [19] and by producing a Matlab/SIMULINK^©^ model of this approach. Matlab^©^ was selected since it allows easy parameter optimisation of any formulated model, and this is a very important capability since some of these parameters in a MBR plant would relate to the combined collective influence of several operational factors that impinge on the fouling mechanisms. Consequently the experimental data and parameter values from the Duclos-Orsello paper [19] were used in the modified model formulation. The solution was slightly different than from the original paper’s results. However by using the SIMULINK^©^ Genetic Algorithm Toolbox, the pore blockage parameter, *α*, the pore constriction parameter, *β*, and the fractional foulant specific cake layer resistance, *f*′*R*′, were made to fit the normalised flow rate data as shown in Figure 1. In Figure 1, the circles represent the measured normalised flow rate values and the lines the fitted dynamic model values using optimised parameters found by the genetic algorithm (GA) routine. Hence this modified model formulation would allow the creation of a plant layout with a practical fouling model that could be validated and calibrated using the real life plant’s own measured data. This is a huge improvement on the original model formulation which was set up only for a laboratory analysis situation.

### 2.4. Scenario Analysis of Model Parameters using Duclos-Orsello’s Original Data

Following verification of the modified model formulation, a scenario analysis was carried out for the three main parameters in this fouling model, namely the pore blockage parameter, *α*, the pore constriction parameter, *β*, and *f*′*R*′ which is the *fractional* foulant layer specific resistance [22]. This scenario analysis was carried out to see which model parameters were most responsive under different simulated operational conditions for a MBR plant. The parameters were analysed under four different bulk concentrations, *C_b_*, ranging from 1 g/L through to 8 g/L. Several simulations were completed for different combinations of parameter values, however for the sake of brevity only some results can be commented on [22]. Table 1 lists three of the most interesting simulations carried out.

**Table 1:** Simulation parameters for scenario analysis.

Simulation No.	*α*	*β*	*f*′*R*′	*R_p_*_0_/*R_m_*
1. equivalent to: *normal MBR operation conditions*	0.0001	0.1	4 × 10^9^	0.5
2. equivalent to: *SMP formation conditions*	0.1	10	4 × 10^10^	0.7
3. equivalent to: *Extreme lab SMP formation conditions*	10	300	4 × 10^11^	0.9

In Simulation 1, the values of *α* and *β* are small with also a low specific resistance of the fraction of foulants on the membrane, *f*′*R*′. Results show there is a progressive almost linear increase in membrane fouling resistance. In Simulation 2, *α* and *β* values are large with a subsequent higher *f*′*R*′ value. Results show that the fouling resistance is very non-linear for high bulk concentrations. Finally, in Simulation 3, *α* and *β* values are extremely large with a subsequent much higher *f*′*R*′ value. Consequently results show a dramatic drop in the flow through the membrane with the fouling resistance being very non-linear for all bulk concentrations.

The outcomes of the scenario analysis seem to indicate that the Simulation 1 situation refers to the normal operational mode of a MBR membrane where effective CiP measures are limiting pore constriction and especially cake build up. Simulation 2 on the other hand is analogous to a situation when soluble microbial product (SMP), the main agent thought responsible for fouling [21], builds up in the MBR reactor due to “stressed” microbial biomass producing it under unusual operational and environmental conditions, e.g., low temperatures, low dissolved oxygen conditions, high salinity of mixed liquor, *etc.* Here the CiP procedures are insufficient to arrest the membrane fouling and more drastic measures such as aggressive chemical cleans are needed to regain flux. Simulation 3 would not occur in practice unless in a laboratory situation since there is a dramatic loss in flux with instant clogging of the membranes by all three fouling mechanisms. However it does prove useful since it shows the model can be used to demonstrate the TMP jump experienced in practice under extreme conditions [21]. In purely physical terms the pore constriction parameter, *β*, clearly governs the intrinsic membrane fouling caused by colloidal and soluble protein material. In the case of a MBR, *β* would relate to the pore size distribution of the membrane, the membrane hydrophobicity, and the aggregation effect of proteins and polysaccharides as SMP [21]. It must be remembered that the typical size of protein macro-molecules, colloids and colloidal aggregates range from nanometres to tens of nanometres whereas the microfiltration membrane pore size range varies from 100 to 200 nanometres. *β* would also slightly depend on the MLSS concentration, as large particles can impede the flow of colloids to the membrane surface and walls. It is worth noting that the majority of the experiments carried out to test the original model were conducted using model protein solutions ranging from concentrations of 1 g/L up to 8 g/L with no solids in the solution to impede pore constriction effects. Usually in a MBR the MLSS is much higher but is composed of mainly particulates while the SMP concentration varies from 100 to say 500 mg/L depending on microbial “stress” conditions. Hence the experimentally measured fouling rate is significantly higher than would ever be experienced in a real plant.

The parameter *α* refers to cake build up and in the scenario analysis includes for the effects due to the CiP mitigation measures and MLSS concentrations effects. This physically means the bulk concentration of solids in the solution and the types and extent of CiP measures would directly influence the *α* value. The *α* value would also indirectly include biomass floc size and species diversity, so that if filamentous bacteria predominated then cake build up would be more rapid with subsequent increased *α* values. The combined parameter *f′R*′ could be correlated in real terms to the biofilm growth on the surface of the membrane. This parameter would then be influenced by the biofilm properties such as adhesiveness, thickness and porosity, and as well as the membrane surface roughness.

Consequently this modified fouling model [22] has parameters that can be directly related to the processes occurring in a typical MBR. However it would still need to be modified to match local MBR configurations and hydrodynamic operational conditions as detailed below.

## 3. Reformulation of Duclos-Orsello Model for Specific MBR Plant Layouts

### 3.1. Further Modifications to Duclos-Orsello Model

This modified fouling model has yet to include the hydrodynamic effects within the MBR plant and the membrane filtration unit that are used in this study to generate the data needed to test this modified model formulation. Also a closer correlation with the main culprit thought by a lot of researchers to cause increased foulability, namely SMP, can be included in the model. This means inclusion of the following effects for specific plant:

1. Membrane surface scour which occurs in both submerged and cross flow sidestream MBR plant. In submerged plant this is usually a constant air scouring and sparging by coarse bubble aeration. For cross flow sidestream plant this membrane scouring is induced by applying a constant cross flow velocity which is developed from continuous recirculation of mixed liquors from the bioreactor (as is the case with the pilot MBR plant described further below). Thus a membrane scour term needs to be added to the model formulation.
2. A backwash mode which is used to remove material accumulated within the membrane by reversing the permeate flow rate. This is usually for a fixed periodic interval of constant duration and intensity (as is the case with the pilot membrane filtration unit described further below). Hence a backwash mode with clean membrane area reset should form part of the model.
3. As mentioned already, most researchers agree that the pore constriction mechanisms within a MBR are probably due to the building up of SMP [21]. This correlation can be catered for in this model by replacing the bulk concentration term, *C_b_*, with a new term, *S_SMP_*, which relates solely to SMP effects.
4. One final reformulation of the model needs to be done to allow the model to be used for varying TMP operational conditions such as occurred with pilot membrane filtration unit mentioned later on in this work.

Hence in a bid to make this model more practical and usable for a typical MBR plant situation, the generalised Duclos-Orsello [19] approach was extensively modified under this study [23]. 

### 3.2. Membrane Surface Scour Effects Inclusion

The following model equations are altered to cater for these specific hydrodynamic effects. In Equation (9), an additional term for scour is added to reduce the cake build up accordingly. Again in this case *C_b_* refers to MLSS of a MBR. This additional term, shown in Equation (12), models the cake removal process, and consists of the cake resistance, *R_p_*, multiplied by a constant removal term, *k_p_* (which itself is dependent on the air sparging rate or cross flow velocity).



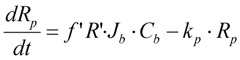
(12)


This removal term is appropriate since a similar formulation was used in the Liang’s MBR membrane fouling model [24], and further this model formulation was calibrated and validated by Janus *et al.* [25]. In this Liang model [24], the change in reversible fouling is given in Equation (13) where *R_r_* is the reversible fouling due to cake build up, *a* is the specific cake resistance, *k_r_* is the fouling strength factor, and *J* is flux through the membrane. Now it can be seen this is identical to the Duclos-Orsello reformulated Equation (12), where *R_r_* is equivalent to *R_p_*, while the term *a* is equivalent to *f*′*R*′, and *k_r_* equates to *k_p_*. In Janus *et al.* [25], this Liang formulation is calibrated and validated on the same set of data as used in this paper under Section 4.5. The experimental data proved very good when compared to the simulation results [25].



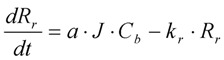
(13)


In Equation (14), an additional term for scour is added to model the increase in the unblocked area of the membrane. This additional term which is the blocked area reset after a backwash interval, *A*_0*(afterBW)*, _multiplied by an area constant term, *k_b_* (which is a function of air sparging rate or cross flow velocity), allows unblocked area to increase with increased scouring rate.



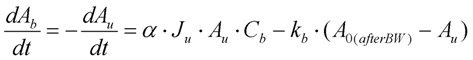
(14)


### 3.3. Backwashing

Since both the pilot MBR plant and the membrane filtration unit are backwashable, this needs some representation in the model such as carried out by Broekmann *et al.* and Busch, *et al.* [8,9]. Unlike Broekmann *et al.* [8], where a dynamic backwash is used, the backwash here is simply modelled by resetting of cake resistance and blocked membrane area by a specifiable amount after the backwash step has been completed. This reset can be altered to cater for full cake and membrane area recovery or only partial recovery. For simplicity’s sake, it is assumed that changing between normal operation and the backwash mode occurs instantaneously. Two additional equations are added to the formulation to cater for this specific hydrodynamic effect. Equation (15) simply resets the cake resistance by a certain amount after the backwash step has occurred. This reset can be altered to cater for full membrane recovery or for complete membrane clogging as described by Lee *et al.* [12].



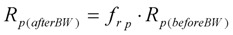
(15)
where *f_rp_* = 1 means no recovery (complete clogging); *f_rp_* = 0 means full recovery. Usually a figure of 0.05 is used to model the gradual build up of irreversible fouling resistance which can only be removed by a chemical clean.

Equation (16) simply resets the blocked area by a certain amount after backwash has occurred. This reset can be altered to cater for full membrane area recovery or for complete membrane area surface covering.



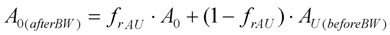
(16)
where *f_rAU_* = 0 means no recovery; *f_rAU_* = 1 means full recovery. Usually a figure of 0.95 is used to model the gradual build up of irreversible fouling resistance which can only be removed by a chemical clean.

### 3.4. SMP Effects Inclusion

In Equation (17), the *C_b_* term which refers to MLSS is changed into the concentration of SMP. This then directly takes into account the effect of the sludge water properties on pore constriction [21].



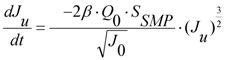
(17)


### 3.5. Total Flow through Membrane for Constant Flux/Varying TMP Operation

This further reformulation allows data to be used which was collected under varying TMP with constant flux stepping conditions. It is worth remembering that since over 90% of MBRs operate in constant flux mode as they are submerged systems [6], then this model reformulation should be capable of handling this situation.

Equation (6) for the blocked flow, *Q_b_*, is reformulated as follows into Equation (18).



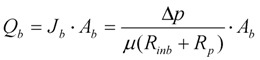
(18)


Rearranging and simplifying Equation (18) gives Equation (19) as below.



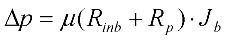
(19)


Differentiating both sides, with *µ* taken as a constant and setting Δ*p* = *TMP*, gives Equation (20).



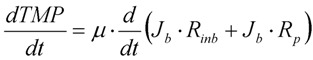
(20)


Then using the Product Rule (Leibniz’s Law) to find the derivatives of the products of these functions gives Equation (21).



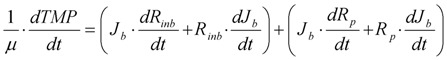
(21)


This can be simplified to Equation (22).



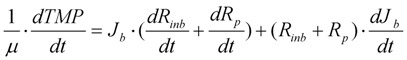
(22)


Thus, Equation (22) allows the TMP to be a variable value for a constant flux, and replaces Equation (6) in the model formulation procedure.

## 4. Model Calibration and Validation—Pilot MBR Plant and Pilot Membrane Filtration Unit

### 4.1. Pilot MBR Plant

The constant TMP/varying flux version of this fouling model was calibrated and validated on data obtained from flux stepping tests performed on an Aquabio Ltd. pilot MBR plant located in Worcestershire that treated salad wash water as industrial effluent. Its basic operational information is described in Table 2 below.

**Table 2:** Operational data for pilot MBR plant.

Aquabio Pilot MBR Plant-sidestream cross flow configuration	
Membrane type and area	Vertical “Berghof” tubular; PVC-C 0.02 μm pore size; 4.1 m^2^
Membrane data	55 tubes each of 8 mm ℘; outer diameter of module is 90 mm
Membrane feed flow (m^3^/h)	10 × *ν* where cross flow velocity is *ν* (m/s)
Feed-Permeate differential pressure	−30 to +600 kPa
Pressure drop along module (kPa)	2.1 × *ν* × *L* where module length is *L*(m) = 3010 mm
Backwash/cleaning regime	Automated backflush possible of varying length (but in flux stepping tests a manual backflush of 120 s was used); periodic hypochlorite clean every few weeks
Biological feed data	COD ~ 700 mg O_2_/L; TSS ~ 50 mg/L
Bioreactor operational data	MLSS ~ 7,000–12,000 mg/L; SMP ~ 500 mg/L

### 4.2. Pilot Membrane Filtration Unit

The constant flux/varying TMP version of this model was next checked by calibrating it on data obtained from flux stepping tests performed on an ITT Sanitaire Ltd. pilot membrane filtration unit. This unit treated tertiary effluent from Cardiff’s sequence batch reactor (SBR) wastewater treatment plant, and its basic operational information is described in Table 3 below.

**Table 3:** Operational data for pilot membrane filtration unit.

ITT Sanitaire membrane filtration unit (without bioreactor)	
Membrane type and area	Horizontal “Kolon” fibres; PVDF 0.1 μm pore size; 20 m^2^
Recirculation flow; permeate flow; backwash	1–2.4 m^3^/h; 0.6–1 m^3^/h; 1.2–1.8 m^3^/h
Backwash interval & duration	Every 4 min with 30 s ON
TMP	300–500 mbar
Aeration rate	13 N m^3^/h from coarse bubble tube diffuser
Cleaning regime	hypochlorite dosed 4 times daily into permeate tank
Feed flow biological data	COD concentration 50 mg O_2_/L; TSS concentration 25 mg/L
Indicative feed flow SMP data	Measured glucose concentration 5 mg/L; measured protein concentration 100 mg/L

### 4.3. Model Calibration Results for Pilot MBR Plant—Fit for 5 Flux Steps at Constant TMP/Varying Flux Regime

**Figure 2:**
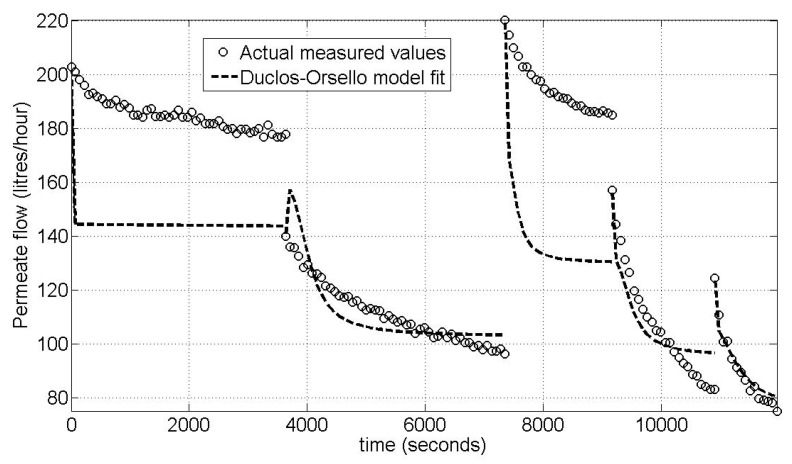
Pilot MBR plant—Modified Duclos-Orsello model fit for 5 flux steps calibration.

Figure 2 shows the result when fitting the model using the calculated optimal parameter sets for five flux steps. The optimal parameter set was determined by running a generic GA procedure in Matlab^©^. As can be seen, the model fit is extremely poor when attempting to fit the data from all five flux steps simultaneously.

### 4.4. Model Calibration Results for Pilot MBR Plant—Fit for Single Flux Step at Constant TMP/Varying Flux Regime

**Figure 3:**
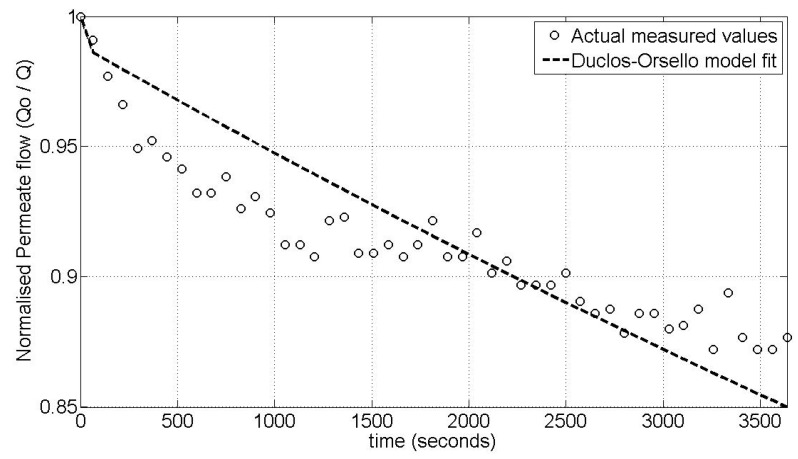
Pilot MBR plant—Modified Duclos-Orsello model fit for single flux step calibration.

In a bid to improve the fit it was assumed that each flux step solution was unique. This could be hypothesized since each flux step with subsequent backwash was actually carried out manually by shutting down the plant, and reversing the flow as necessary whilst also manually altering the membrane module throttle valve setting which itself significantly altered the hydrodynamics occurring within the tubular arrangement. This assumption means that the data set used was actually discontinuous in time between individual flux steps, and therefore each step should be considered separately by the model on an individual data-by-data basis. This altered model optimization procedure was tried to ascertain if a better fit could be achieved. Figure 3 is the result obtained. It is clear that the model fit improves when flux steps are taken individually as unique solutions. Also the fit improves when the specific step regime produces fluxes and TMPs that are well below critical conditions so that the membrane performance is not compromised. However it must be realized that during normal plant operation, the throttle valve settings would be kept fixed, and these settings are only being changed here to facilitate the flux stepping procedures, so that the model behavior can be tested across the full flux range from sub-critical through to supra-critical ranges.

### 4.5. Model Validation Results for Pilot MBR Plant—Fit for Single Flux Step at Constant TMP/Varying Flux Regime

**Figure 4:**
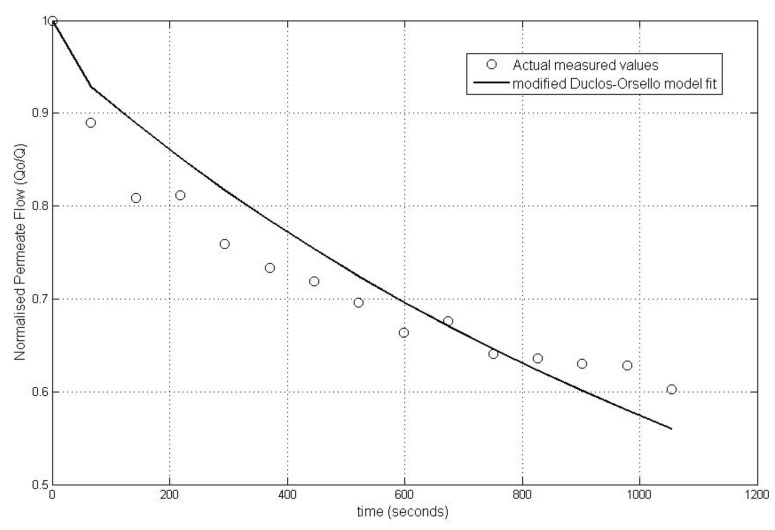
Pilot MBR plant—Modified Duclos-Orsello model fit for single flux step validation.

This model formulation was validated by using data taken from a later single flux step coupled with the same parameter values used for the calibration step. The fit under this validation procedure is depicted in Figure 4.

### 4.6. Pilot Membrane Filtration Unit—Fit for 8 Flux Steps at Constant Flux/Varying TMP

**Figure 5:**
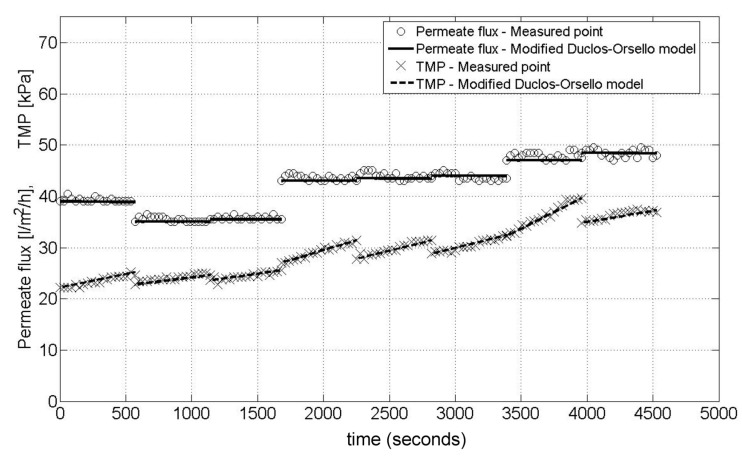
Pilot membrane filtration unit—best Modified Duclos-Orsello model fit for 8 flux steps.

Figure 5 shows the result obtained when using the calculated optimal parameter sets for eight flux steps for this second pilot unit. The model fit is extremely good which in this case can be attributed to the following reasons:

- The membrane unit has no complex bioreactor (*i.e.*, no significant biological and biochemical variations to be considered).
- Very low mixed liquor concentrations and subsequent very low SMP levels gave an extremely consistent membrane performance.
- The plant flow train is simple. Also the entire flux stepping procedure is automated including the backwash procedure, meaning it is less prone to human error as compared to manually altering a throttle valve setting.
- Other factors that influenced these exceptional results are this was a constant flux operation giving simpler hydrodynamics with no discontinuities in time between flux steps.
- Also the plant had been operating consistently over a long period of time unlike the Aquabio pilot MBR plant. Further, the flux stepping tests all occurred on the same day, and also the air sparging procedure used to clean the membrane was at a very high rate (*i.e.*, much higher than for a full size commercial unit) and occurred continuously even during the backwashes. This meant extreme membrane clogging was very unlikely.

### 4.7. Summarising Model Fitting Results for Both Sets of Data from the Pilot Units

Table 4 compares the GA fit for all the model runs carried out with a very low GA final fit number near to zero indicating a very good fit. The GA fit number is calculated automatically by the GA procedure in Matlab^©^ and refers to the values of the best and mean score of the population at every generation in the GA run. The next generation of the population is computed using the fitness of the individuals in the current generation. Thus the GA fit numbers referred to in Table 4 are the GA final fit numbers determined at the end of the GA run. 

It is clear for the Aquabio pilot MBR plant, that the fit is very good when the flux steps are taken individually as unique solutions. Also the fit improves when the specific step regime produces fluxes and TMPs that are well below critical conditions, so that the membrane performance is not compromised and does not have a knock-on effect on the subsequent flux step. In comparison the ITT membrane unit gives results even better than the best Aquabio pilot MBR plant result. This is as expected due to the factors already discussed in the previous section.

**Table 4:** Comparison of GA final fit numbers for various plants for different flux step combinations modelled.

Plant type and flux step combo	Best GA fit	Mean GA fit
Aquabio—all 5 flux steps	72.1059	75.9315
Aquabio—single flux step	1.9425	2.2955
ITT pilot unit Cardiff—all 8 flux steps	0.037944	1.0377

When comparing the optimised parameter values shown in Table 5 that were determined by the GA routine, a clear pattern again emerges. The pore blockage parameter, *α*, is high for all modelled data sets like the situations in the scenario analysis described as Simulations 2 and 3 when the SMP formation conditions predominate and it is not a normal MBR scenario (see Section 2.4). This is as expected because most of the flux steps are well above the normal operating range of the membranes under consideration. The pore constriction parameter, *β*, is of the same order as occurred in the scenario analysis procedure in Section 2.4 which is as expected. However the *f*′*R*′ parameter which is the product of fractional amount of total foulant contributing to deposit growth, *f*′, and the specific protein layer resistance, *R*′, is much reduced when compared to the scenario analysis values. This can be explained since the scenario analysis used pure protein solutions such as bovine serum albumin whilst the real life plant have mixed liquors of which only a small proportion consist of protein matter.

The *R_p_*_0_/*R_m_* parameter is of the same order for all flux steps as those determined under the previous scenario analysis. The four remaining parameters which refer to the extensive model reformulation to include membrane scour affects and backwash regimes cannot be compared. However the membrane scour terms, namely *k_p_* and *k_b_*, appear to be of a sensible size, and the size of the backwash terms *f_rp_* and *f_rAU_* appear to make sense as well. The *f_rp_* parameter value is quite high for most flux steps meaning that full recovery of the membrane is never achieved which is as expected for a membrane being stepped under fluxes beyond its normal operating range. Also, the *f_rAU_* parameter value is quite low for most flux steps meaning that full recovery of the membrane unblocked area after a backwash is never achieved which is as expected for a membrane being flux stepped beyond its normal operating range.

In summary, this means most of parameter values are of the same order as stated in the Duclos-Orsello paper [19], or for those new parameters they are of a size that makes theoretical and mathematical sense. Consequently this model formulation does appear accurate enough to be used to model a membrane filtering mixed liquors and experiencing subsequent fouling and clogging.

**Table 5:** Comparison of optimal parameter values for various plants for different flux step combinations modelled.

Plant type and flux step combo	*α*	*β*	*f*′*R*′	*R_p_*_0_/*R_m_*	*k_p_*	*k_b_*	*f_rp_*	*f_rAU_*
Aquabio—all 5 flux steps	6934	0.07	9184	0.17	43.50	12.50	0.98	0.013
Aquabio—single flux step	5237	1.80	6909	0.66	83.94	359	0.53	0.73
ITT pilot unit Cardiff—all 8 flux steps	3469	0.14	8079	0.56	183	694	0.97	0.26

## 5. Conclusions

Overall it is clear that the phenomenological model performed very well even though it took a considerable time to be developed into a useful format, and the model had to be calibrated and validated using complex GA routines and procedures. It could be argued that this modified model itself may be slightly over parameterized and a simple linear regression would give a decent fit under the normal operation range of the MBR (e.g., sub-critical fluxes). However, a linear regression model could not accommodate the TMP jump when it occurs at supra-critical fluxes, while this fouling model should be able to express all of this behavior as it has been calibrated and validated on flux stepping data which is well above the membrane manufacturer’s normal operating TMP/flux ranges. There are occasions when the TMP/flux ranges do go above recommended levels during normal MBR operation. However this happens occasionally and usually only for short periods, and any model should be able to cater for this. Conversely in comparison to the comprehensive Broeckmann *et al.* [8] and Busch *et al.* [9] models, this one only has a limited number of parameter sets and hence reduced degrees of freedom. Therefore it is felt this fouling model version strikes the right balance between the number of parameters within it; its ability to mimic the full range of TMP/fluxes; its basis on classical fouling mechanisms; and its general useability and flexibility for operation and control. In conclusion, most of the optimized model parameter values are of the same order as stated in the original Duclos-Orsello paper [19]. For those new parameters created in the modified model they are of a size that makes theoretical and mathematical sense although this still needs to be tested using further data sets. Consequently this model formulation does appear to be accurate enough to be used to model a real life membrane or MBR system filtering mixed liquors and experiencing subsequent fouling and clogging events.

## Nomenclature

*C_b_*: Bulk concentration (g/L)
*f*′: Fractional amount of total foulant contributing to deposit growth
*J_0_*: Initial flux rate of clean membrane (m/s)
*Q_t_*: Total volumetric flow rate (m^3^/s)
*Q*_0_: Initial volumetric flow rate (m^3^/s)
*R_m_*: Resistance of the clean membrane (m^−1^)
*R_p_*_0_: Original resistance of the deposit (m^−1^)
*R*′: Specific protein layer resistance (m/kg)
*R_p_*: Resistance of the deposit (m^−1^)
*t*: Filtration time (s)
*t_p_*: Filtration time after initial membrane blocking occurs(s)
Δ*p*: Constant total trans-membrane pressure (Pa)

## Greek letters

*a*: Pore blockage parameter (m^2^/kg)
*β*: Pore constriction parameter (kg)

## Membrane surface scour terms

*k_p_*: Membrane surface scour constant term related to cake resistance
*k_b_*: Membrane surface scour constant term related to unblocked area
*A_0(afterBW)_*: Original unblocked area after backwash interval (m^2^)

## Backwash mode terms

*R_p(afterBW)_*: Cake resistance after backwash interval (m^−1^)
*R_p(beforeBW)_*: Cake resistance before backwash interval (m^−1^)
*f_rp_*: Cake resistance recovery constant term
*A*_0_: Original area before backwash commences (m^2^)
*A_U(beforeBW)_*: Unblocked area before backwash interval (m^2^)
*f_rAU_*: Unblocked area recovery constant term

## SMP term

*S_SMP_*: Concentration of SMP in the sludge water (g/L)

## Variable TMP term

Rate of change of Δ*p*

## Liang membrane fouling model

*R_r_*: Reversible (cake) resistance (m^−1^)
*a*: Specific cake resistance (m/kg)
*k_r_*: Fouling strength factor (m/kg)
